# Case of Pulmonary Extramedullary Hematopoiesis Responding to Ruxolitinib

**DOI:** 10.1016/j.lrr.2022.100290

**Published:** 2022-02-07

**Authors:** Ayumi Fujimoto, Shunichi Hamaguchi, Ritsuro Suzuki

**Affiliations:** a Department of Hematology, Shimane University Hospital, Izumo, Japan; b Department of Internal Medicine, Division of Medical Oncology and Respiratory Medicine, Shimane University Faculty of Medicine, Izumo, Japan

**Keywords:** Pulmonary Extramedullary Hematopoiesis, Myelofibrosis, Ruxolitinib, Case report

## Abstract

We report a case of 43-year-old woman diagnosed with essential thrombocythemia in 1992. She was diagnosed with secondary myelofibrosis in 2011. Later, she suffered mild dyspnea, which gradually worsened. She was admitted to our hospital to evaluate the cause in 2014. Chest computed tomography showed ground-glass opacity (GGO) in the lungs. A lung biopsy revealed various hematopoietic cells, including abnormal megakaryocytes, infiltrating the alveolar septum, suggesting pulmonary extramedullary hematopoiesis. She was successfully treated by ruxolitinib and her disease is well controlled for more than 7 years. To keep this phenomenon in mind when see the patients with dyspnea is important.

## Case presentation

**Figure 1:**
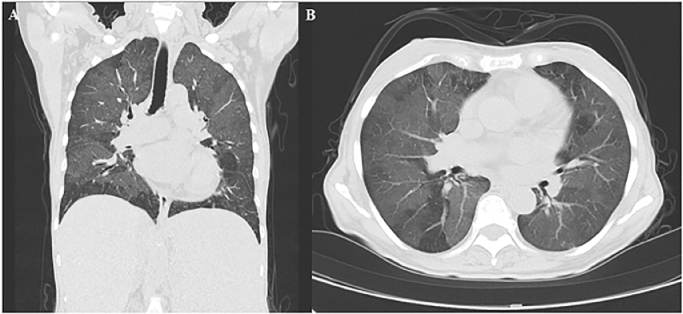
Chest computed tomography images of the patient with pulmonary-extramedullary hematopoiesis at diagnosis. (A) Coronal section and (B) horizontal cross-section. Both images show diffusely extended ground-glass opacity in the lungs.

**Figure 2:**
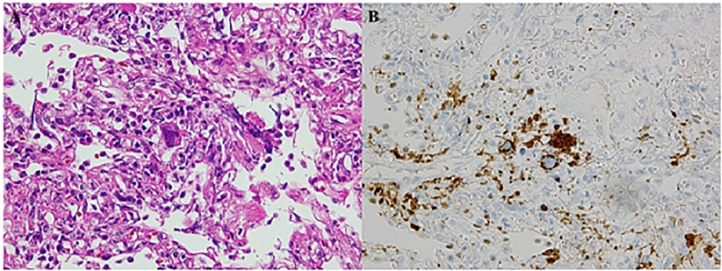
Immunohistochemical findings of lung biopsy. (A) hematoxylin and eosin stain (magnification x400) and (B) CD42b antibody stain (magnification x400). Both images show abnormal megakaryocytes infiltrating in the alveolar septum.

A 43-year-old Japanese woman with a history of hypertension, hyperlipidemia, and hyperuricemia was referred to our hematological service because of thrombocythemia and splenomegaly in 1992. Laboratory studies showed white blood cell count of 11,400/µL, hemoglobin level of 13.3g/dL, and platelet count of 1170 × 10^9^/L. A diagnosis of essential thrombocythemia (ET) was made by bone marrow aspiration and biopsy. The patient was initially treated with oral busulfan. Because her splenomegaly worsened, the treatment was changed to hydroxycarbamide (HU) in 2001. The patient's disease was well controlled for a decade. However, an asymptomatic GGO with 7 mm diameter was unexpectedly observed in the left upper lobe of the lung by computed tomography (CT) in 2011. Leukocytosis with immature myeloid series also gradually developed from 2013. White blood cell count elevated from 20,000/µL in 2013 to 30,000/µL in 2014. Her splenomegaly gradually worsened and extended to 6 cm below the umbilicus in 2014. These findings suggested that she developed secondary myelofibrosis (MF). Bone marrow biopsy revealed MF and *JAK2 V617F* mutation later. At the same time, the patient suffered mild dyspnea, and the symptom gradually worsened and impaired her daily activities. Physical examination revealed mild fine crackles in the lungs and systolic heart murmur. Laboratory studies revealed the following: abnormal complete blood count including white blood cell count of 25,400/µL (with 4.6% blasts, 0.5% promyelocytes, 2.0% myelocytes, 4.6% metamyelocytes, 13.7% banded neutrophils, 51.8% segmented neutrophils), hemoglobin level of 7.9g/dL, and platelet count of 29 × 10^9^/L^4^; normal renal function; lactate dehydrogenase level of 1,514 U/L. The levels of antibodies associated with autoimmune diseases, including antinuclear antibody and anti-neutrophil cytoplasmic antibody, were within the normal range. Her arterial blood gas revealed a pH of 7.39, partial pressure of arterial CO2 of 35 mmHg, partial pressure of oxygen (PaO2) of 68 mmHg, and bicarbonate level of 21.2 mmol/l. Pulmonary function tests showed obstructive lung disease, with forced expiratory volume in 1 s (FEV1) of 65%, a ratio of FEV1 to forced vital capacity (FVC) (FEV_1.0%_) of 72.2% (Tiffeneau index), a vital capacity (VC) of 68% of the predicted value, and a diffusing capacity for carbon monoxide (DLCO) of 59% of the predicted value. Echocardiography showed an ejection fraction of 55%, tricuspid regurgitation peak gradient (TRPG) of 43 mmHg, right ventricular systolic pressure (RSVP) of 53 mmHg, and pulmonary artery end-diastolic pressure (PAEDP) of 20 mmHg, suggesting pulmonary hypertension (PH). Chest CT showed that the previously detected GGO, diffusely extended to her lungs with a mosaic pattern to her lungs, without evidence of pulmonary embolism (Figure 1A-B). The symptoms further worsened. She felt dyspnea even when walking at home. She finally required home oxygen therapy for low oxygen levels in her blood. Transbronchial lung biopsy was conducted to investigate the cause of her lung disease. The lung specimen showed septal thickening and various hematopoietic cells, including abnormal megakaryocytes that were positive for CD42b (Figure 2A-B) and factor VIII, myeloid series, and a few blasts that were positive for c-kit, in the alveolar and alveolar septum. A diagnosis of pulmonary extramedullary hematopoiesis (P-EMH) was made. Ruxolitinib (20 mg bid/day) was administered instead of HU. Subsequently, her dyspnea was dramatically improved only one week after treatment initiation. One month later, dyspnea was almost disappeared. She did not feel dyspnea even when running. Splenomegaly also significantly improved to 6cm below the left costal margin 2 months after treatment initiation. Chest CT showed that the GGO in the lungs was almost disappeared 3 months after treatment initiation. Then, the GGO in the lungs finally disappeared 8 months after the initiation of ruxolitinib. The pulmonary function tests showed improved results with an FEV of 91%, a ratio of FEV_1.0%_ of 76%, VC of 90% of the predicted value, and DLCO of 64% of the predicted value. PH also improved, with a TRPG of 25 mmHg, RSVP of 35 mmHg, and PAEDP of 13 mmHg, re-evaluated by echocardiography. She is asymptomatic and has recovered her ability to perform daily activities. Splenomegaly also improved to 3 cm below the costal margin. Her disease has been well-controlled with ruxolitinib for more than 7 years.

## Discussion

EMH refers to the presence of hematopoiesis outside the bone marrow and is also called as “myeloid metaplasia”. EMH is observed in many conditions including myeloproliferative neoplasms such as myelofibrosis and chronic myeloid leukemia, and chronic hemolytic anemia such as thalassemia. The pathogenesis of EMH is not well understood. It is assumed that several factors, including cytokines, chemokines, adhesion molecules, and proteases would favor the proliferation and mitigation of stem cells from the bone marrow, resulting in ectopic hematopoiesis. Although the most commonly involved sites of EMH are the liver and spleen, any sites can be involved. Para-vertebral regions are relatively common sites for intrathoracic EMH, while the lungs are uncommon. P-EMH was first identified by autopsy in 1912 [1]. Approximately 50 cases of P-EMH described in the literature, and has been reported to correlate with the progression of primary disease [2]. The most commonly involved site of EMH in the thorax is posterior mediastinum, that is, para- or intra-vertebral sites. Patients with paravertebral EMH are generally asymptomatic. In contrast, patients with P-EMH in the alveolus, alveolar septum, pleura, and pleural effusion are not commonly seen; these patients have symptoms of dyspnea, cough, and, less frequently, chest pain [2]. Chest CT is the most reliable modality for detecting P-EMH. However, the images are widely dispersed, and there is no standard or specific CT image for P-EMH. Previous reports have shown a variety of images, including pulmonary nodules or masses, alveolar septal thickening, GGO, and patchy consolidation [2], [3], [4], [5], [6]. The recommended process of diagnosis and treatment is not well known.

The CT image in the present case showed diffusely extended bilateral GGO, which correlated with the worsening exertional dyspnea. The decreased PaO2 and DLCO, in addition to echocardiographic PH mimicked the presence of pulmonary fibrosis or interstitial pneumonia. However, a surgical biopsy of the lung tissue made it possible to make a correct diagnosis of P-EMH. The pulmonary function tests of P-EMH show obstructive lung disease and low diffusing capacity [4, 7], which were consistent with the current case. The prevalence of PH in patients with MF is 14-56% in the literature [8, 9]. There exist several hypotheses for the pathogenesis of PH in patients with MF, including increased platelet-derived growth factor resulting in smooth muscle hyperplasia, pulmonary capillary obstruction by megakaryocytes and platelets, aberrant *JAK/Src-STAT3* signaling, and pulmonary vascular remodeling by myeloid cells. P-EMH itself is also associated with PH [10]. In the current case, based on the results of echocardiography, mean pulmonary arterial pressure (mPAP) was estimated to exceed 25 mmHg, suggesting the presence of PH. After the treatment with ruxolitinib the PH resolved as P-EMH relieved, suggesting that PH was one of the manifestations of P-EMH.

The historical treatment of P-EMH, such as cytoreductive agents including HU, busulfan, or anagrelide had limited effects. In contrast, low-dose whole-lung radiation was efficacious as a palliative treatment in several reports [11, 12]. Ruxolitinib, a JAK2 inhibitor, was reported to be associated with the development of PH and worsen exertional dyspnea in a patient with *JAK2 V617F* mutation-positive MF. In this case, the symptom of dyspnea improved after the cessation of ruxolitinib, suggested drug-induced PH [13]. Another report presented a patient with polycythemia vera (PV) harboring *JAK2 V617F* mutation who developed exertional dyspnea six months after the initiation of ruxolitinib [6]. In this patient, the development of PH coincided with worsening leukocytosis, suggesting that ruxolitinib was not effective for the treatment of PV. By contrast, Maccaferri M, et al. reported that ruxolitinib was effective for the treatment of P-EMH in a patient with *JAK2 V617F* mutated MF. The clinical symptoms, such as fatigue, cough, and dyspnea was relieved, and radiological findings of GGO and splenomegaly also improved [7]. Ruxolitinib was also effective for PH in patients with MF [14]. In our patient, ruxolitinib successfully improved patient symptoms, pulmonary function, radiological findings of the lungs, PH, and splenomegaly. In addition, ruxolitinib was also reported to be effective for primary MF patients with EMH in the kidney. Ruxolitinib is an effective candidate for the treatment of symptomatic MF patients with EMH, although further evidences are required to ascertain the efficacy of this agent.

In summary, we presented a case of P-EMH accompanied by secondary MF with confirmation of lung biopsy. Although P-EMH is rare, this complication should be highlighted to provide an accurate diagnosis and appropriate treatment.

## Author contributions

A.F. wrote the paper and R.S. revised the paper. All author reviewed the manuscript.

## Declaration of Competing Interest

A.F. and S.H. has no financial conflicts of interest to disclose. R.S. received honoraria from Bristol-Myers Squibb, Novartis, Kyowa Hakko Kirin, Chugai Pharmaceuticals, Takeda, Meiji Seika Pharma, MSD, Otsuka, Celgene, Eisai Pharmaceuticals, Abbvie Inc., and Janssen, outside the submitted work.
